# O22 Pregnancy planning in non-criteria antiphospholipid syndrome

**DOI:** 10.1093/rap/rkab067.021

**Published:** 2021-10-19

**Authors:** Qurat Ul Ain Amjad, Spencer Ellis

**Affiliations:** Rheumatology, Lister Hospital, East and North Hertfordshire Trust, Stevenage, United Kingdom

## Abstract

**Case report - Introduction:**

Antiphospholipid Syndrome (APS) is an autoimmune condition manifested by vascular thrombosis with or without pregnancy related morbidities. APS classification criteria require detection of persistent antibodies: Anti-Cardiolipin Antibody (aCL), Anti-Beta Glycoprotein I (ab2GPI) and circulating Lupus Anticoagulant (LAC).

Criteria for the diagnosis of obstetric antiphospholipid syndrome allow for optimal treatment planning in pregnancy and reduction of foetal and maternal morbidity. There are, however, several “non-criteria” features of APS, including late pre-eclampsia, which may also infer adverse obstetric outcomes.

We highlight a treatment dilemma in a young female with lupus and APS with history of previous pregnancy that was complicated by pre-eclampsia.

**Case report - Case description:**

We describe a case of a 26-year-old female with systemic lupus erythematosus (SLE), “triple positive” APS markers (aPL) and non-criteria APS obstetric morbidity.

During her first pregnancy she presented at 22 weeks with severe and frequent migraine episodes. She had no other active clinical manifestations of SLE. There was no prior history of pregnancy and therefore no previous obstetric morbidity nor had she had any vascular thrombotic episodes. She had been commenced on low-dose aspirin (LDA) at the start of her pregnancy and was already taking hydroxychloroquine (HCQ). The dose of HCQ was increased to 400mg daily leading to improvement in the headaches. She had persistent strong ACL antibodies, positive LAC and ab2GPI antibodies. ANA was positive with hypocomplementemia and neutropenia but normal range double-stranded DNA antibodies.

After discussion with a tertiary centre, it was recommended to continue HCQ and LDA. Low molecular weight heparin (LMWH) was not proposed as she did not fulfil criteria for obstetric APS. Her pregnancy was subsequently complicated by pre-eclampsia and post-partum sepsis.

Two years later she sought advice to plan her second pregnancy, raising important questions as to whether the same treatment strategy should be followed or if alternative approaches should be considered, given the history of pre-eclampsia and its association with APS. Again, she did not meet the international consensus (revised Sapporo) criteria for diagnosis of obstetric APS.

After further discussion with the tertiary centre, she commenced high-dose aspirin 150mg/day alone. Uterine artery dopplers were recommended and LMWH proposed if bilateral notching was noted. Dopplers were normal.

Her blood pressure (BP) fluctuates between 130-145mmHg systolic. She has occasional crops of mouth ulcers but no other SLE features. Migraines continue every 2—3 weeks although severe attacks are infrequent. C4 is persistently low with normal Ds DNA and urine protein/creatinine ratio.

**Case report - Discussion:**

Our case raises questions on how pregnancy should be safely managed in patients without a prior history of APS-related pregnancy complications but who nevertheless have risk factors, in the form of APS antibody positivity and non-criteria obstetric manifestations of APS. Her previous pregnancy was treated with LDA and HCQ which had proved insufficient in avoiding the outcome of pre-eclampsia.

The objective of prophylactic treatments is to prevent adverse outcomes such as miscarriage, which carry significant maternal morbidity including psychological manifestations such as depression and anxiety. In first pregnancies, mothers have no existing obstetric history, such that current treatment guidance provides few recommendations for preventative interventions, even where other risk factors are apparent. Furthermore, non-criteria manifestations of obstetric APS are now well recognised, which may further highlight potential risk for obstetric morbidity but fail to trigger interventions as they are not included within the current international criteria. These manifestations include late pre-eclampsia, placental abruption and late pregnancy loss, unexplained *in vitro* fertilisation failures, two unexplained miscarriages and three non-consecutive miscarriages. Low positive antibody titres and intermittent aPL are also included.

Studies suggest that women with non-criteria APS may benefit from standard treatment for obstetric APS which includes LMWH in addition to aspirin, with good pregnancy outcomes.

HCQ has a dose-dependent effect in preventing thrombosis through inhibition of platelet aggregation and aPL Ab-related thrombosis. However, pregnancy itself is a prothrombotic condition and in the presence of persistent triple positive aPL is a risk factor for adverse pregnancy outcomes.

Further studies are needed to define the risk of non-criteria APS on pregnancy outcomes and define optimal treatment strategies.

**Case report - Key learning points:**

1. Obstetric APS carries significant risk for maternal foetal morbidity including miscarriage and pre-term birth.
2. Significant maternal psychological effects are recognised even following a single miscarriage.
3. The international consensus (revised Sapporo) criteria for diagnosis of obstetric antiphospholipid syndrome exclude certain non-criteria clinical and laboratory manifestations that may confer adverse pregnancy outcomes.
4. First pregnancies are challenging to risk stratify in patients with identified aPL as there will be no prior pregnancy history to reference.
5. Ultrasound doppler velocimetry is a useful non-invasive tool to diagnose poor placental perfusion, a predictor of obstetric complications, hence identifying high-risk pregnancies.
6. Further research is required to establish the diagnostic validity, predictive value, and treatment implications of non-criteria manifestations of obstetric APS.

